# Lung Infection Due to Species Infrequently Isolated in the Community Setting: Klebsiella variicola and Acinetobacter baumannii

**DOI:** 10.7759/cureus.110904

**Published:** 2026-06-15

**Authors:** Mauro Geller, Mendel Suchmacher, Aline Sitnoveter, Rafael Nigri

**Affiliations:** 1 Department of Immunology, Universidade Federal do Rio de Janeiro (UFRJ), Rio de Janeiro, BRA; 2 Department of Immunology, Centro Universitário Serra dos Órgãos (UNIFESO), Teresópolis, BRA; 3 Department of Immunology, Instituto de Pós-Graduação Carlos Chagas, Rio de Janeiro, BRA; 4 Internal Medicine Residency, University of Central Florida College of Medicine, Orlando, USA; 5 Department of Internal Medicine, New Jersey Medical School, Newark, USA

**Keywords:** acinetobacter baumannii, antimicrobial effectiveness, diagnosis, klebsiella variicola, lung infection

## Abstract

The authors report a case of pulmonary infection caused by two typically nosocomial bacterial agents, *Klebsiella variicola* and *Acinetobacter baumannii*, simultaneously evidenced in a 65-year-old male patient from the community. Both diagnoses were established after culture and matrix-assisted laser desorption/ionization time-of-flight mass spectrometry (MALDI-TOF MS) analysis of sputum, and the patient was successfully treated with oral levofloxacin, with microbiological eradication subsequently confirmed by three negative follow-up sputum cultures. The singularity of this case challenges the conventional paradigm distinguishing community- and hospital-acquired infections and underscores the importance of maintaining a high index of suspicion for atypical pathogens in routine clinical practice.

## Introduction

From the earliest phases of medical training, clinicians are taught to distinguish between so-called community-acquired and hospital-acquired bacterial agents [1]. Diagnostic and therapeutic decisions are shaped by the bacterial species expected in each environment, the patient profile, and the clinical picture. Community-acquired respiratory infections are typically associated with agents such as *Streptococcus pneumoniae*, *Haemophilus influenzae*, and *Mycoplasma pneumoniae*, whereas hospital-acquired infections are more commonly linked to organisms such as *Pseudomonas aeruginosa*, methicillin-resistant *Staphylococcus aureus*, and *Acinetobacter* species. *Klebsiella variicola* and *Acinetobacter baumannii* are clinically significant, predominantly opportunistic pathogens, both capable of multidrug resistance and both more often encountered in healthcare settings than in the community.

However, this rule of thumb is not always observed. In some instances, two pathogenic “hospital” species can be simultaneously identified where they would not be expected. We describe a case in which two typically nosocomial species were isolated from the same community-originated patient and discuss the diagnostic and conceptual implications of this finding.

## Case presentation

A 65-year-old man presented in December 2021 with acute non-productive cough, nasal congestion, progressive asthenia, and an axillary temperature of 37.8°C (100°F). He received symptomatic treatment only, with no antibiotics prescribed at that stage. After seven days, the cough progressed to clear sputum production, asthenia worsened, and the axillary temperature rose to 38.4°C (101.1°F). A nasopharyngeal swab for SARS-CoV-2 antigen was negative.

The patient had a history of upper left lung adenocarcinoma, treated by surgical resection in 2019; he received no chemotherapy or radiotherapy, and the remaining lung tissue preserved its function. He also had paroxysmal bronchospasms since 2008, for which he used inhaled fluticasone/vilanterol intermittently during occasional acute episodes rather than as continuous long-term therapy. He denied smoking and reported no recent hospital admission or outpatient antibiotic exposure before the onset of symptoms.

A laboratory workup was performed in December 2021 (Table 1). An automated sputum culture was obtained, and species-level identification was performed by matrix-assisted laser desorption/ionization time-of-flight mass spectrometry (MALDI-TOF MS) on the cultured isolates, yielding *K. variicola* and *A. baumannii*, with the antimicrobial susceptibility profiles shown in Table 2.

**Table 1:** Laboratory workup results.

Parameter	Result	Reference range
Hemoglobin	16.0 g/dL	12.5-17.5 g/dL
White blood cell count	15,250 cells/mcL (1,220 bands; 12,047 polymorphonuclears; 915 lymphocytes; 1,068 monocytes)	4,000-11,500 cells/mcL
Platelet count	245,000/mcL	150,000-450,000/mcL
Erythrocyte sedimentation rate	26 mm/h	< 20 mm/h
C-reactive protein	3.9 mg/dL	< 1.0 mg/dL
Blood urea nitrogen	14 mg/dL	7-20 mg/dL
Creatinine	0.9 mg/dL	0.7-1.3 mg/dL

**Table 2:** Antimicrobial susceptibility of the sputum isolates.

Klebsiella variicola	Acinetobacter baumannii
Sensitive: amikacin, ampicillin/sulbactam, cefepime, ceftazidime, ceftriaxone, ciprofloxacin, ertapenem, gentamicin, imipenem, levofloxacin, meropenem, piperacillin/tazobactam, sulfamethoxazole/trimethoprim.	Sensitive: amikacin, gentamicin, imipenem, meropenem. Intermediate sensitivity: levofloxacin.

Given the susceptibility profiles and the availability of an oral formulation, levofloxacin was prescribed empirically at 750 mg daily for 14 days. Clinical improvement was observed by day three of therapy, and the patient completed the full course. Follow-up sputum cultures obtained at 7, 15, and 30 days after completion of therapy showed no growth of either *K. variicola* or *A. baumannii*, confirming microbiological eradication and sustained clinical recovery (Table 3).

**Table 3:** Follow-up sputum culture results after completion of levofloxacin therapy.

Timepoint	Klebsiella variicola	Acinetobacter baumannii
7 days post-treatment	No growth	No growth
15 days post-treatment	No growth	No growth
30 days post-treatment	No growth	No growth

## Discussion

*Klebsiella variicola*

*K. variicola* is a Gram-negative, nonmotile, facultatively anaerobic rod belonging to the *Klebsiella pneumoniae* complex within the family *Enterobacteriaceae*. It causes infections of the bloodstream, respiratory tract, and urinary tract, typically in debilitated individuals with conditions such as autoimmune disease, malignancy, or alcoholism - a profile inconsistent with the present patient. There are no semiotic parameters by which *K. variicola* infection can be clinically distinguished from *K. pneumoniae* infection. The species is intrinsically resistant to ampicillin owing to its beta-lactamase activity but is generally sensitive to agents of other antibiotic classes. Of clinical concern, roughly half of hospital isolates are extended-spectrum beta-lactamase (ESBL) or carbapenemase producers, a trait associated with the higher mortality reported for *K. variicola* relative to *K. pneumoniae* [2,3].

Because purely phenotypic methods are unreliable for *K. variicola* identification, molecular, genomic, and proteomic techniques are warranted to avoid misclassification as *K. pneumoniae*. In the present case, identification was achieved by MALDI-TOF MS; it should nonetheless be acknowledged that even mass spectrometric methods can have reduced discriminatory power within the *K. pneumoniae* complex. For ESBL non-producing strains amenable to outpatient management, oral sulfamethoxazole-trimethoprim is a practical option, whereas most other recommended agents require parenteral administration [3].

Acinetobacter baumanii

*A. baumannii* is a Gram-negative, non-fermentative, aerobic, nonmotile, catalase-positive coccobacillus that accounts for approximately 80% of infections attributed to the *Acinetobacter* genus. It belongs to the *A. baumannii* complex and has been isolated from humans, soil, and vegetables. The organism forms biofilms and can persist on moist or dry surfaces for weeks. Recognized risk factors for colonization include residence in a nursing home, intensive care admission, and prior exposure to third-generation cephalosporins, fluoroquinolones, or carbapenems - none of which applied to the present patient [3,4].

*A. baumannii* is one of the ESKAPE pathogens, reflecting its capacity to acquire antimicrobial resistance from other organisms, and approximately half of isolates are resistant to therapy ordinarily indicated for Gram-negative species. Community-acquired *A. baumannii* pneumonia occurs chiefly during warm, humid seasons in tropical countries and can follow a fulminant course, with purulent or blood-stained cough, fever, pleuritic chest pain, respiratory failure, and septic shock; lobar consolidation is the characteristic radiographic finding. Recognized predisposing factors - alcohol consumption, diabetes mellitus, and chronic lung disease - were again absent in this patient. As with *K. variicola*, no clinical parameters reliably distinguish *A. baumannii* infection from infection by other *Acinetobacter* species, and distinguishing clinical infection from simple colonization can itself be challenging [4,5].

Although community-onset *A. baumannii* does not display the aggressive resistance profile of hospital strains, agents commonly used for community-acquired infections, such as ceftriaxone and the fluoroquinolones, are frequently inactive against it. There is no consensus outpatient regimen; oral agents with documented activity include amoxicillin-clavulanate, ciprofloxacin, and trimethoprim-sulfamethoxazole [5,6]. In the present case, the favorable clinical response, together with three negative follow-up sputum cultures, supports a diagnosis of true infection rather than colonization and confirms eradication with the levofloxacin regimen despite the isolate’s intermediate susceptibility to that agent.

Limitations

This report has limitations inherent to its outpatient context. The patient was managed entirely in the home setting, where samples were collected; consequently, chest imaging, complete vital sign documentation, auscultation findings, blood cultures, and bronchoalveolar lavage were not obtained. Microbiological follow-up was, however, available: three sequential sputum cultures obtained at 7, 15, and 30 days after completion of therapy showed no growth of either pathogen, documenting eradication and supporting genuine infection rather than colonization. Although the patient lacked the classic risk profile for nosocomial pathogens, his prior lung adenocarcinoma treated by surgical resection and his intermittent use of inhaled corticosteroids may have produced subtle alterations in local pulmonary defenses that cannot be entirely excluded as contributing factors. Species identification relied on automated culture with mass spectrometry (MALDI-TOF MS); the recognized limitations of these methods in discriminating *K. variicola* from *K. pneumoniae* and within the *A. baumannii* complex are acknowledged.

## Conclusions

To the best of our knowledge, this is an infrequently reported instance in which *K. variicola* and *A. baumannii*, both commonly regarded as hospital opportunistic pathogens, were simultaneously identified in an otherwise healthy patient from the community. The case underscores the importance of maintaining a high index of suspicion for atypical presentations in clinical practice so as to avoid the diagnostic pitfalls inherent in established epidemiological paradigms. Outpatient treatment guidance for community-onset infection by these organisms remains a gap in the literature, and future case series and genomic analyses of community-acquired isolates would help clarify their epidemiology and optimal management.
